# Calling at the highway: The spatiotemporal constraint of road noise on Pacific chorus frog communication

**DOI:** 10.1002/ece3.2622

**Published:** 2016-12-20

**Authors:** Danielle V. Nelson, Holger Klinck, Alexander Carbaugh‐Rutland, Codey L. Mathis, Anita T. Morzillo, Tiffany S. Garcia

**Affiliations:** ^1^ Department of Fisheries and WildlifeOregon State UniversityCorvallisORUSA; ^2^ ARCS Foundation ScholarArcataCAUSA; ^3^ Bioacoustics Research ProgramCornell Lab of OrnithologyCornell UniversityIthacaNYUSA; ^4^ Cooperative Institute for Marine ResourcesOregon State University and NOAA Pacific Marine Environmental LaboratoryNewportORUSA; ^5^ Department of Integrated BiologyOregon State UniversityCorvallisORUSA; ^6^ Department of Natural Resources and the EnvironmentUniversity of ConnecticutStorrsCTUSA

**Keywords:** acoustic, amphibian, anthropogenic, communication, road noise, spatiotemporal

## Abstract

Loss of acoustic habitat due to anthropogenic noise is a key environmental stressor for vocal amphibian species, a taxonomic group that is experiencing global population declines. The Pacific chorus frog (*Pseudacris regilla*) is the most common vocal species of the Pacific Northwest and can occupy human‐dominated habitat types, including agricultural and urban wetlands. This species is exposed to anthropogenic noise, which can interfere with vocalizations during the breeding season. We hypothesized that Pacific chorus frogs would alter the spatial and temporal structure of their breeding vocalizations in response to road noise, a widespread anthropogenic stressor. We compared Pacific chorus frog call structure and ambient road noise levels along a gradient of road noise exposures in the Willamette Valley, Oregon, USA. We used both passive acoustic monitoring and directional recordings to determine source level (i.e., amplitude or volume), dominant frequency (i.e., pitch), call duration, and call rate of individual frogs and to quantify ambient road noise levels. Pacific chorus frogs were unable to change their vocalizations to compensate for road noise. A model of the active space and time (“spatiotemporal communication”) over which a Pacific chorus frog vocalization could be heard revealed that in high‐noise habitats, spatiotemporal communication was drastically reduced for an individual. This may have implications for the reproductive success of this species, which relies on specific call repertoires to portray relative fitness and attract mates. Using the acoustic call parameters defined by this study (frequency, source level, call rate, and call duration), we developed a simplified model of acoustic communication space–time for this species. This model can be used in combination with models that determine the insertion loss for various acoustic barriers to define the impact of anthropogenic noise on the radius of communication in threatened species. Additionally, this model can be applied to other vocal taxonomic groups provided the necessary acoustic parameters are determined, including the frequency parameters and perception thresholds. Reduction in acoustic habitat by anthropogenic noise may emerge as a compounding environmental stressor for an already sensitive taxonomic group.

## 1 Introduction

Sound is four‐dimensional and influenced by multiple parameters at once (Vehrencamp & Bradbury, 2012): it expands in three spatial dimensions outward (the active space) and also exists for a finite amount of time, the fourth dimension. This necessitates that the vocalizations produced by communicating animals will also be four dimensional, having three spatial and one temporal dimension (henceforth termed “spatiotemporal communication”). However, most studies on calling anurans in noisy environments, as well as other taxa exposed to anthropogenic noise, analyze the acoustic features of the vocalizations as well the calling behavior (source level, call duration, call rate, and frequency) independently (e.g., Cunnington & Fahrig, 2010; Halfwerk, Lea, Guerra, Page, & Ryan, 2015; Lengagne, 2008; Parris, Velik‐lord, & North, 2009). Rarely are all features considered in a way that intuitively describes the spatiotemporal communication for a species. It is important to examine not only the spatial but also the temporal dimensions of animal communication, as both are important factors in communication (Vehrencamp & Bradbury, 2012).

Anthropogenic noise levels are rising worldwide, to the detriment of wildlife (Barber, Crooks, & Fristrup, 2010; Clark et al., 2009). Anthropogenic noise causes background interference that affects the perception of sound, a phenomenon known as masking, in which long‐range communication is significantly hindered by background noise (Bee & Swanson, 2007; Lohr, Wright, & Dooling, 2003; Read, Jones, & Radford, 2014). Many taxa have been found to respond to high levels of background noise by changing their vocalizations in ways that decrease masking. Increasing source level (changing signal amplitude or getting louder) in the face of noise masking, termed the Lombard effect (Zollinger & Brumm, 2011), has been observed in response to masking in songbirds (e.g., Brumm & Todt, 2002), cetaceans (e.g., Holt, Noren, Veirs, Emmons, & Veirs, 2009), bats (e.g., Hage, Jiang, Berquist, Feng, & Metzner, 2013), and several other vertebrate species (Zollinger & Brumm, 2011). Changing the temporal aspect of calls (Bee & Schwartz, 2013), such as altering the duration of the call or how often the call is produced, can also reduce masking by shifting spatiotemporal communication to avoid overlap with noise. Additionally, acoustic species can change their frequency (commonly described as pitch) (McGregor, Leonard, Horn, & Thomsen, 2013), which has been described in birds, anurans, and other species (Naguib, 2013). However, in many of these species, it is unclear how much noise may degrade overall habitat quality (Cunnington & Fahrig, 2010; Sun & Narins, 2005). It is possible to estimate the active space for a given calling animal, and this has been done for several vocal species. For example, Gall, Ronald, Bestrom, and Lucas (2012) calculated the effect of urbanization on active space of the brown‐headed cowbird (*Molothrus ater*) and found that the sound propagation depended on both the phase of the song and the surrounding environment. For anurans, the active space of the spring peeper was calculated by Parris (2002) in relation to its environment and calling position. However, these models are primarily concerned with the spatial component of vocalization and neglect any effect temporal calling patterns may have on the active space and time of a signal.

Anthropogenic noise pollution can degrade habitat and be a significant stressor for many vocalizing organisms, including many frog species (order: Anura) (Barber et al., 2010). The source of most anthropogenic noise stress for anurans is road noise; frogs will change their vocalizations in response to high levels of road noise (Cunnington & Fahrig, 2010; Lengagne, 2008; Sun & Narins, 2005). Halfwerk et al. (2015) found evidence supporting the Lombard effect in calling túngara frogs, which increased source levels in high‐noise situations. Several examples of temporal changes, either in rate or duration, have also been found in vocal anurans in response to high levels of noise, effectively minimizing the temporal overlap with noise in the same frequency band (Kaiser & Hammers, 2009; Lengagne, 2008; Sun & Narins, 2005). Further, frequency shifts above that of anthropogenic noise have been observed in a few species of frogs, altering the space over which the vocalization can be perceived (Cunnington & Fahrig, 2010; Parris et al., 2009). It remains unclear the extent to which these modifications compensate for masking for an individual frog (McGregor et al., 2013; Vélez et al., 2013). Anthropogenic noise many not be the primary stressor for calling amphibians, which are the most threatened vertebrate group in the world (Beebee & Griffiths, 2005; Stuart, 2004), but it is an added layer of stress that can have significant consequences to anuran populations in highly modified landscapes. Spatiotemporal communication models could more accurately assess the impacts of anthropogenic noise on population dynamics and community structure.

**Figure 1:**
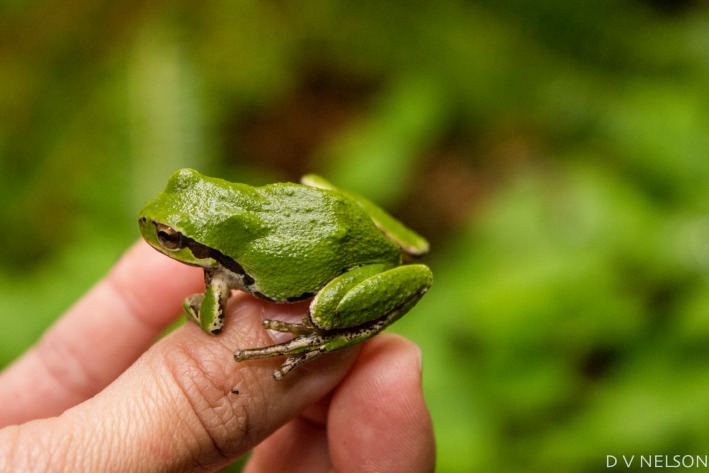
The study species, *Pseudacris regilla*, or Pacific chorus frog

The purpose of this study was twofold. First, we tested the predictions that the vocalization (call) parameters of the Pacific chorus frog (*Pseudacris regilla*, Figure 1), a common species in the northwestern United States, would vary across a traffic noise gradient and secondly that vocalization parameters would lead to a quantifiable difference in the spatiotemporal communication for this species. Specifically, we predicted that the parameters of frequency would shift upward above the bandwidth of noise containing the most energy and that call rate and call duration would be negatively correlated and change depending on noise level, but that source level would not change. Additionally, we predicted that there would be changes in these parameters based on temperature, because anurans are ectothermic and their energy expenditure is highly correlated with temperature (Aenz, Itzgerald, & Aum, 2006). Second, we developed a simplified spatiotemporal communication model that can be easily extensible to other species and habitats. To test our predictions, we compared the vocalizations of Pacific chorus frogs across breeding sites experiencing a range of traffic noise conditions. To create a spatiotemporal hemispherical model of a frog's communication space that accounted for calling behavior of males on floating vegetation in the water to females that may be in the water or on vegetation above it, we took average values of vocalization parameters and background traffic noise and used spherical spreading models to determine the volume of space over which the vocalizations can be perceived. As the lower bands of the Pacific chorus frog vocalization are overlapped by the spectrum of road noise (Figure 1), this species is particularly at risk of being masked by road noise.

## 2 Methods and Materials

### 2.1 Field methods

**Figure 2:**
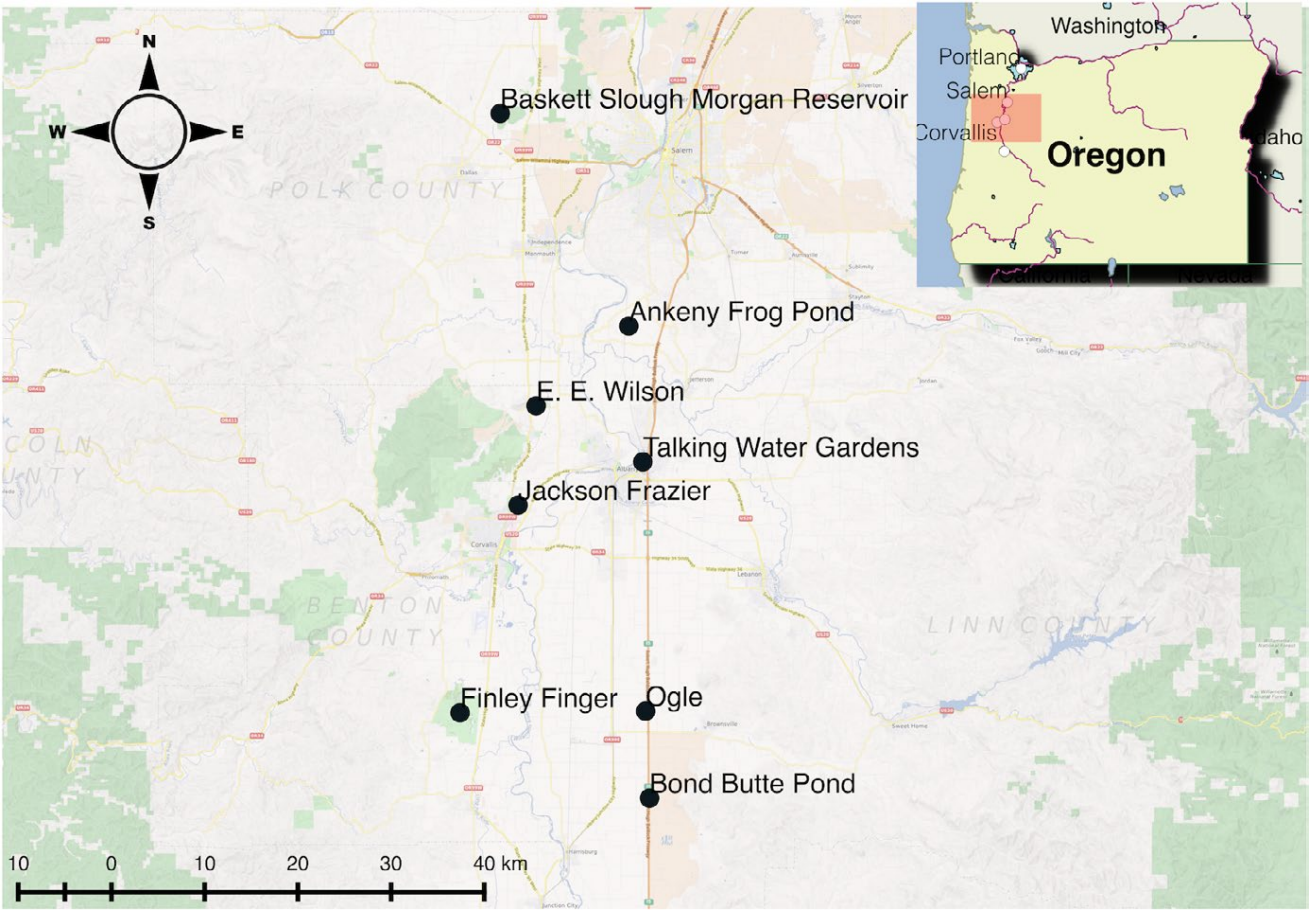
Sites used for analysis

We selected eight sites to acoustically monitor across a gradient of road noise (i.e., distance from roads experiencing greater than 30,000 annual average daily traffic; Oregon Dept. of Transportation). Monitoring and sampling were conducted over 2 years (2014–2015) during two consecutive Pacific chorus frog breeding seasons (February–May). Meteorological data were taken from the weather station (FNWO3) in nearby William L. Finley National Wildlife Refuge, Corvallis, OR, to account for temperature effects (Figure 2). While microhabitat temperature fluctuations are possible, we chose to use a broader measure of temperature to reflect the broader impact of noise across a landscape rather than at a microhabitat scale.

### 2.2 Soundscape monitoring

We characterized the anthropogenic and Pacific chorus frog components of each site's soundscape over the course of both breeding seasons by quantifying ambient road noise levels and timing as well as general chorus structure. Passive recorders (WildlifeAcoustics Songmeter models SM1, SM2, and SM2+ with two microphones recording in stereo; microphone sensitivity −35 ± 4 dB re 1 V/Pa for SM1, −36 ± 4 for SM2 and SM2+) were installed at all sites (Table 1, Figure 2) and left in place for the duration of the breeding season. Battery changes and data storage downloads were performed biweekly to ensure continuity in recording. Recorder sampling rate was set at 44.1 kHz with 16‐bit resolution, producing recordings with a frequency range of 0–22,050 Hz. Gain settings varied by recorder (Table 1).

**Table 1:** List of sites, years recorded, gain settings, number of frogs (*n*), and number of recording nights (nights when directional recording took place)

Site name	Years recorded	Songmeter gain settings (2014, 2015, in dB)	Number recorded (2014, 2015)	Recording nights (2014, 2015)
Bond Butte	2014, 2015	42 through 4/3, 36 till removal; 42	7, 11	2, 3
Ogle Rd	2015	N/A, 36	N/A, 14	N/A, 4
Talking Water Gardens	2014	42, N/A	6, N/A	1, N/A
Finley Finger Pond	2014, 2015	42, 51	6, 13	2, 5
Jackson Frazier Wetland	2014, 2015	42, 51	6, 11	2, 2
E. E. Wilson	2015	N/A, 51	N/A, 11	N/A, 3
Ankeny Wood Duck Pond	2015	N/A, 51	N/A, 10	N/A, 3
Baskett Slough Morgan Reservoir	2015	N/A, 51	N/A, 10	N/A, 3

Passive acoustic recording schedules in 2014 were based on the timing of sunset due to the crepuscular nature of calling in this species (Allan, 1973). Recordings began one hour before sunset and continued for 4 hrs total. This schedule operated daily and tracked sunset based on location and corresponding seasonal changes in photoperiod. In 2015, the recording schedule was extended to 8 hrs, from 4 p.m. to midnight daily (February 1–April 5) and then from 4 p.m. to 2 a.m. daily (April 6—end of breeding chorus activity). This was done to attempt to capture the end of the chorus each night, but was unsuccessful because chorusing persisted beyond 2 a.m. The change in recording time has no effect on any of the results presented here as chorus timing was not examined in this study. Additionally, ambient noise levels were extracted from the beginning of recording days (2014: 1 hr before sunset; 2015: 4 p.m.) to prevent confounding the ambient noise levels with chorusing frog levels. As this section of Interstate 5 does not have a rush hour, this constitutes an accurate assessment of the road noise levels at each site. Recorder time was not changed to compensate for daylight savings time; therefore, all recordings were made in Pacific Standard Time.

### 2.3 Call structure monitoring

To assess call structure, directional recording was completed at four sites in 2014 and at seven sites in 2015. Eight unique sites in total were used. We opportunistically localized individual calling frogs before each directional recording event. The recording equipment was comprised of a Sennheiser MKH20 microphone (sensitivity: −32 dB re 1 V/Pa) in a Telinga parabolic directional housing, attached to a Zoom H4n recorder with SD storage. Single‐channel recordings at a sampling rate of 44.1 kHz with 16‐bit resolution were produced. We took recordings throughout the breeding season in order to account for any possible seasonal effects (e.g., temperature and photoperiod shifts). At least 10 frogs were recorded per site; 105 total frogs were recorded across both years. At least 5 min were allowed to pass between individual recordings to allow surrounding frogs to recover from any disturbance by the observers. Additionally, once a frog was localized, we stopped all observer movement and light disturbance for 5 min before beginning the recording in order to allow the frog to resume normal calling behavior. We recorded individual frogs in situ for three to 5 min and measured the distance from the microphone to an individual frog after recording was complete. This allowed us to later calculate the standard source level at 1 m without having to standardize the recording distance in the field, which may have disturbed calling behavior. We manually adjusted gain settings on the recorder to maximize individual detectability prior to the start of recording and noted them for later reference in calculating source levels. We minimized the probability of recording the same individual more than once per night by consistently walking in one direction around the breeding site. Additionally, we started recording surveys at different locations in an attempt to capture different individuals throughout the breeding season.

### 2.4 Data processing and analysis

#### 2.4.1 Passive acoustic recordings

Data were processed using program SoX (Bagwell, 2013) and a custom‐written R package^1^ that enables batch processing of large audio files. All data were initially processed into single‐channel audio files. Audio files were put through a 1–4.5‐kHz band‐pass filter to highlight any noise within the bandwidth of the Pacific chorus frog call. SoX was used to extract RMS amplitude measures in the frequency band of interest. To determine ambient road noise level without confounded frog chorusing for a given night of directional recording, the RMS amplitude of the files between 4 p.m. and 5 p.m. (before chorusing started) was converted in program R to absolute loudness (dB re 20 μPa) measurements using individual microphone specifications and gain settings. Because loudness is commonly measured on a logarithmic scale (decibel), we took the median (50th percentile) of the amplitude between 4 p.m. and 5 p.m. This time period was chosen because it is very unlikely that there would be a substantial frog chorus in those hours (presunset) during the breeding season (Schaub & Larsen, 1978); additionally, this was verified by visual examination. Therefore, it constitutes an accurate assessment of the ambient road noise levels at each site. To determine the general patterns of low‐frequency traffic noise outside of the bandwidth of the Pacific chorus frog call, a similar procedure was run on a 1–1,000 Hz bandwidth. Measures were extracted for every 15‐min period during the time of recording, 4 p.m.–12 a.m.

#### 2.4.2 Directional acoustic recordings

**Figure 3:**
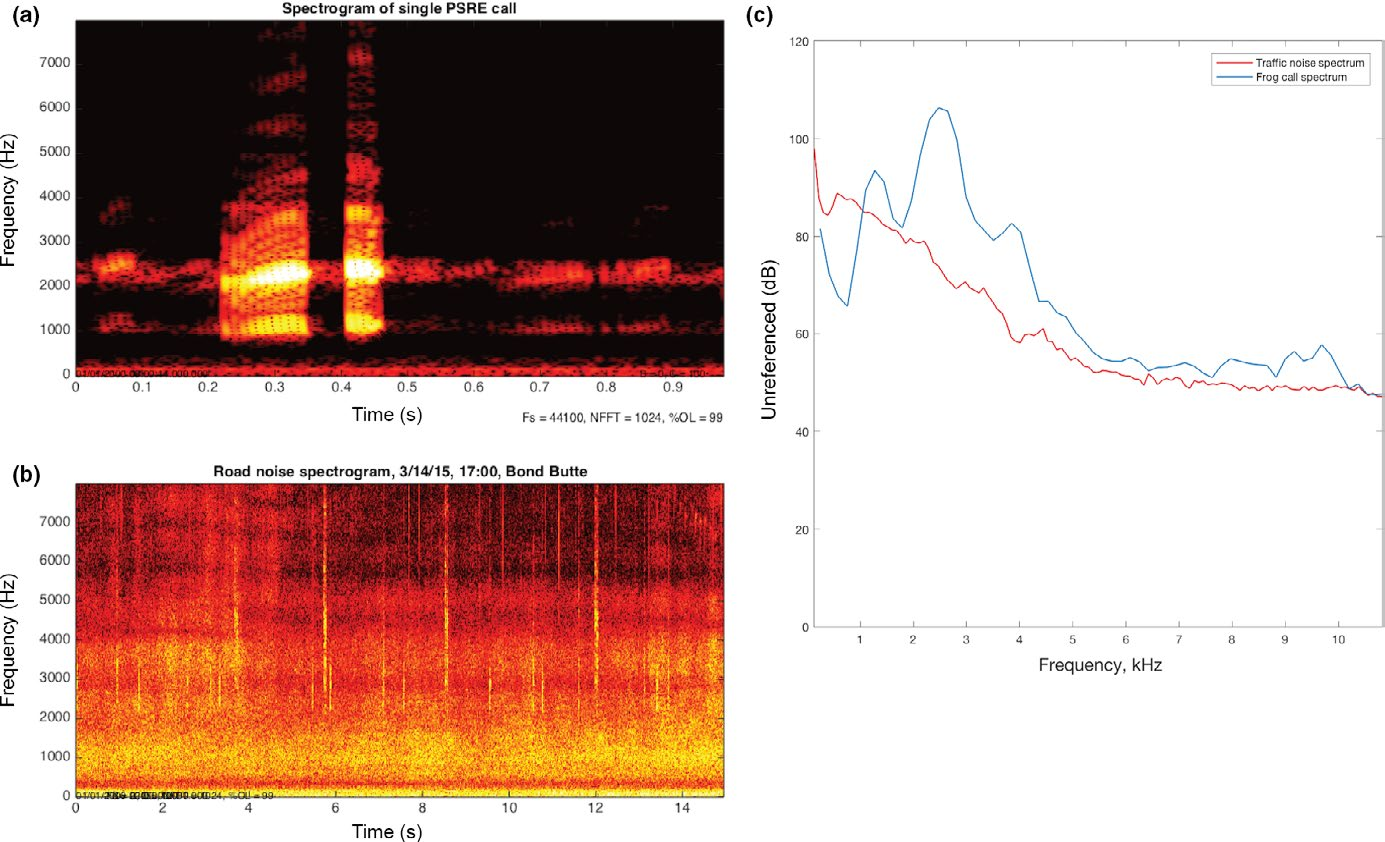
(a) One‐second spectrogram of a single Pacific chorus frog call. (b) Fifteen‐second spectrogram of road noise in overlapping bandwidth of frog call. (c) Power spectra of frog call taken at 1 m (blue) and received traffic level (red)

Before acoustic analysis was conducted, all recordings were downsampled to 11,025 Hz. Spectrograms (graphic representation of frequency, time, and intensity; Figure 3) of each directional recording were generated using Raven Pro 1.5 (Cornell Lab of Ornithology) with 256 point fast Fourier transform (FFT), Hann window, and 50% overlap, and the MATLAB‐based program Osprey (Mellinger & Bradbury, 2007) using the same parameters except a Hamming window. Recordings were manually reviewed, and each call from an individual frog was counted using RavenPro 1.5. Calls selected for further analysis of frequency, time, and source level parameters were those that did not overlap with other individual frog calls, and which had visually and aurally distinctive start and end points and high signal‐to‐noise ratio. One frog recording was excluded because the data were corrupted.

Of the 105 frogs recorded (year 1: *n* = 25; year 2: *n* = 80), 89 frog recordings (year 1: *n* = 20; year 2: *n* = 70) were used for further analysis. Selections were manually drawn on the spectrogram image to encompass the temporal start and end points of each two‐syllable call. To encompass the majority of the energy of each call while excluding most of the low‐frequency road noise, measurements were restricted to the bandwidth 1.0–4.5 kHz. Within this bandwidth, filtered RMS source levels were extracted using SoX and converted in R using the microphone specifications and recorder gain settings to decibel level (dB re 20 μPa). This converted measure was chosen instead of the more traditional sound‐pressure meter because it allows us to examine decibel level only in the bandwidth of the frog call, which is a good proxy for the perception bandwidth of anurans in general (Simmons & Moss, 1985). Centroid frequency measures were taken using a 4,096‐point FFT based on the 3 dB filter bandwidth of 3.87 Hz. Duration measures were taken using a 256‐point FFT. Before calculating the measurements described below, a 0.05‐s buffer was added to either side of the start and end time to account for methods used by the noise‐resistant feature set (Mellinger & Bradbury, 2007).

These selected calls were then analyzed for call parameters (dominant frequency and duration) from the noise‐resistant feature set (NRFS; Mellinger & Bradbury, 2007) within the MATLAB‐based program Osprey. The parameters it measures correspond to more traditional acoustic measurements, but are considered more robust to attenuation and noise conditions. This is particularly important for this study given the range of noise conditions found across the sites depending on proximity to high‐volume roads. Measures used from the NRFS were duration and centroid frequency. Once acoustic analysis was completed for directional recordings, all data were imported into R (R Core Team 2016) for statistical analyses.

Call parameters extracted from the directional recordings for the purposes of this study included band‐limited (1.0–4.5 kHz) RMS source level (dB re 20 μPa), call duration (s), centroid frequency (Hz), and call rate (defined as the number of calls per minute of recording). Analyses were performed at the level of an individual calling male frog; call duration, peak overall frequency, and filtered RMS source level were averaged across all calls for an individual. In addition, temperature measurements that were taken at hourly intervals over each day were also averaged between the hours of 7 p.m. and 10 p.m. for a given night of directional recording. Passive recorder failure occurred on several days throughout each season due to battery failure and/or poor weather conditions. Therefore, only days with complete data were used in noise analyses.

### 2.5 Statistical analysis

To examine how road noise differed between the eight sites, we constructed a linear model that modeled ambient noise levels extracted from the passive recordings against *A* = 1/distance^2^, as predicted by the inverse square law for sound propagation outdoors (Embleton, 1996). Distance was the linear range between the pond site and the nearest high‐traffic road. This variable *A* is based on how sound propagates outdoors and transforms an otherwise exponential response into a linear one.

To examine the effects of road noise and temperature covariates on Pacific chorus frog call structure, we constructed a linear mixed effects model for each parameter of interest. The response variables of interest were source level, frequency, call rate, and call duration for individual frogs. The continuous variables noise and temperature were included as fixed main effects (covariates), and date nested within site was included as a random effect to account for any random error created by breeding site‐level or date‐level differences. Visual inspection of residual plots from the final models did not reveal any obvious deviations from homoscedasticity or normality for the parameters of call rate, mean frequency, and mean source level. Heteroscedasticity was observed for the parameter duration; therefore, the response variable was log‐transformed. All statistical analyses were conducted using R v. 3.3.0 (R Core Team 2016), package *nlme* (Pinheiro & Bates, 2015), and package *lme4* (Bates, Maechler, Bolker, & Walker, 2015).

### 2.6 Spatiotemporal communication model

The loudness of a signal at the receiver in question is known as the received level; in this case, the received level would be the loudness of a male advertisement call at the position of a Pacific chorus frog female at or near the breeding site. A simplified model was created (Equation (1)) of the received level (RL) for individual frogs based on (1) the source levels (*s*) and (2) ambient road noise levels (*n*) across our breeding sites (Embleton, 1996), which were derived from the measures of background noise loudness calculated from the passive recording data. This measure does not take into account the perceptual threshold of this species and should therefore be regarded as a conservative estimate of the masking threshold. While atmospheric absorption can have an effect in other conditions, for our purposes, its effect was negligible (absolute value 0.04 dB). Therefore, it was left out of our final model. Excess attenuation (*A*_e_) was derived from Marten and Marler (1977) by taking the median of excess attenuation terms in the bandwidth of the frog call energy for open field, deciduous forest without leaves, and deciduous forest with leaves to encompass the variable habitat found at each pond. In this case, it was determined to be 0.2 dB/1 m.

(1) $$\text{RL}=s-20\log_{10}r-A_{e}r$$

Using this model of received level, the radius (*r*) at which the received level was attenuated to the point at which it could no longer be heard over the background noise *n* was calculated (when RL = *n*). The radius for an individual frog was modeled against noise and temperature in a linear mixed model. The radius was used to calculate the hemispherical, three‐dimensional volume of the communication space. Additionally, a temporal component was incorporated by using the predicted call rates and durations (*d*) from given temperatures and noise levels based on the statistical models. Using these results, the spatiotemporal communication was determined from a time–volume (m^2^*s) calculation of an individual calling frog for a given minute at the overall mean temperature of 9.59°C (Equation (2)).

(2) $$\text{time.volume}=d*\text{callrate}*\left(\frac{2}{3}\pi r^{3}\right)$$

## 3 Results

### 3.1 Ambient noise levels

**Figure 4:**
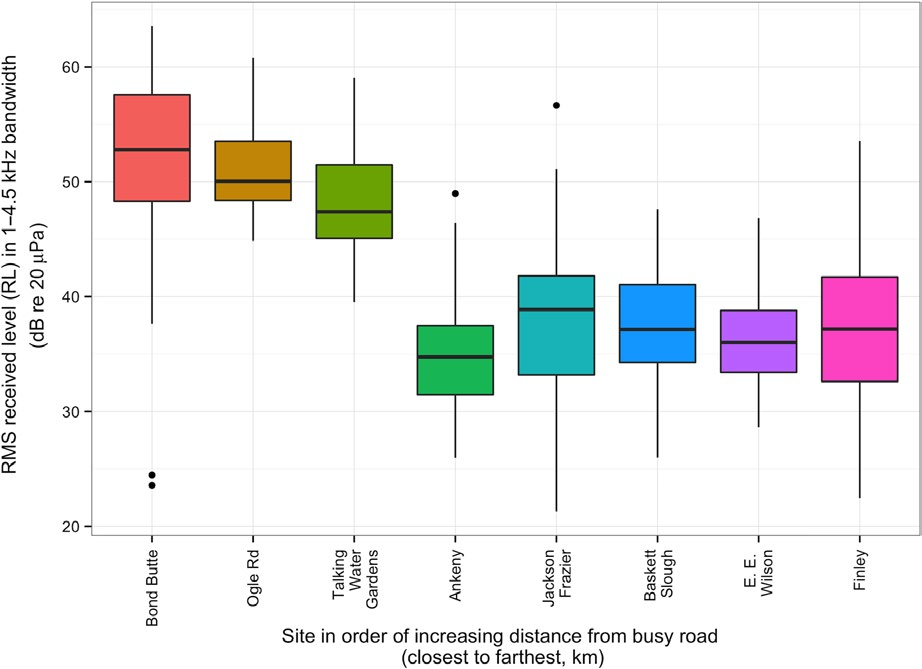
Ambient road noise levels (dB re 20 μPa) per site against distance from busy road (km) (*t*_6_ = −6.33; *p* < .005)

Ambient noise levels in the 0–1,000 Hz range differed among sites, decreasing with increasing distance from a road with 30,000 or more average cars per day. Such roads included encompassed Interstate 5 and some areas of Highway 99 near Salem, OR (Figure 4). When modeled against *A* = 1/distance^2^, as predicted by the inverse square law for sound propagation outdoors (Embleton, 1996), noise levels differed significantly with increasing distance (*t*_6_ = −6.33; *p* < .005). Sites close to busy roads (<1 km) averaged 52.27 dB re 20 μPa (SE: ±0.4714) during the hours of 4–5 p.m. daily, while sites farther from busy roads (>1 km) averaged 37.48 dB re 20 μPa (SE: ±0.6633) during those hours (Figure 4). Ambient noise at sites far from roads was due to various factors such as wind and rain, air traffic flyover, bird vocalization, and vehicular maintenance at the sites. We found no significant seasonal or hourly difference in ambient road noise levels at any of the sites.

### 3.2 Call structure

**Figure 5:**
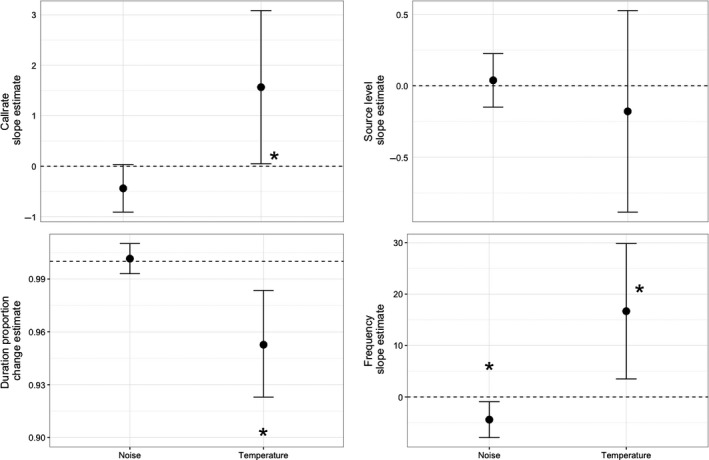
Confidence intervals of slope estimates (duration: proportional change) for each parameter of interest. * indicates significant (p < 0.05) difference from zero

Pacific chorus frogs did not change source level in response to different temperatures (*t*_89_ = −0.528, *p = *.604) or differing levels of noise (*t*_89_ = 0.431, *p* = .672, Table 2, Figure 5). Centroid frequency of Pacific chorus frog calls was found to be significantly related to both ambient road noise level and temperature. For every 1°C increase in temperature, centroid frequency is estimated to increase 16.676 Hz (*t*_89_ = 2.641, *p* = 0.016, Table 2, Figure 5). This response in call frequency is weakly mediated by anthropogenic noise in the opposing direction, such that for every 1 dB increase in noise level, centroid frequency is estimated to decrease 4.422 Hz (*t*_89_ = −2.646, *p* = .016, Table 2, Figure 5). However, this shift fails to be biologically significant, as its maximum still falls within the range of the standard deviation for this parameter. There was a significant relationship between duration of calls and temperature. For every 1°C increase in temperature, duration is estimated to decrease by approximately 5% (*t*_89_ = −3.180, *p* = .005, Table 2, Figure 5). There was no significant relationship between duration of calls and ambient road noise level (*t*_89_ = 0.381, *p* = .708, Table 2).

**Table 2:** Final linear mixed models for each parameter

	Dependent variable				
	Call rate (calls/min)	Frequency (Hz)	Log (duration in s)	Source level (dB re 20 μPa)	Radius (m)
Median noise level	−0.438*p* = .067*	−4.422*p* = .016**	0.002*p* = .708	0.039*p* = .672	−0.498*p* = .000***
Temperature	1.566*p* = .044**	16.676*p* = .016**	−0.048*p* = .005***	−0.179*p* = .604	−0.094*p* = .583
Observations	89	89	89	89	89

*p < .1, **p < .05, ***p < .01.

There was also a significant relationship between the mean call rate and temperature. For every 1°C increase in temperature, call rate is estimated to increase 1.566 calls per minute (*t*_89_ = 2.154, *p* = .044, Table 2, Figure 5). While the relationship between mean call rate and noise was not statistically significant, there was a trend toward a slight decrease in call rate with increasing levels of noise (*t*_89_ = −1.941, *p* = .067).

### 3.3 Spatiotemporal communication

The four‐dimensional spatiotemporal communication is reduced for an individual Pacific chorus frog at sites with relatively high noise levels. In the bandwidth of the frog call, a significant reduction in the radius of spatiotemporal communication was found (*t*_89_ = −12.656, *p* < .005, Table 2, Figure 5). The radius of spatiotemporal communication was found to be reduced by 0.498 m for every 1 dB increase in background road noise levels. When this reduction was calculated into the volume measurements, it corresponded to a 1.04 m^3^ per dB reduction in the volume of spatiotemporal communication. Therefore, even without taking into account the downward trend in call rate, there was an overall reduction in the time–volume of communication by several orders of magnitude at the loudest noise levels observed, compared to the quietest area.

## 4 Discussion

We provide the first assessment of the effects of ambient road noise on the call structure of the Pacific chorus frog, a vocalizing anuran. We predicted that frogs would change some aspects of their call structure (source level, call rate, duration, and frequency) depending on the level of noise at their breeding site. Source levels did not change with increasing levels of road noise, which supports other evidence that the Lombard effect is not present in many species of anurans. We predicted that Pacific chorus frogs would change the temporal structure of their calls in response to noise and that call rate and call duration would be negatively correlated. This prediction was not upheld, such that call duration did not change in response to noise. We predicted that frogs would shift the dominant frequency of their vocalizations upward to prevent masking by road noise. Contrary to our predictions, frequency was slightly downshifted. We also predicted that temperature would impact these vocalization parameters. Higher temperatures increased call rate and mean centroid frequency and decreased call duration. However, temperatures did not vary enough across sites within the breeding season to elicit a significant difference in vocal parameters. For a given temperature, it was feasible to compare sites across a gradient of noise exposures. Thus, we have demonstrated that frogs at noisy ponds do not vocalize differently than frogs at quiet ponds in a biologically significant way; that is, they do not change their vocalizations in a way that helps to mitigate the effects of masking.

Males that communicate within the bandwidth of ambient road noise should experience selective pressure to adjust their call parameters to differentiate their signal from background noise containing the most energy (Vélez et al., 2013). Hypothetically, this may be accomplished by shifting the frequency of the vocalization such that it no longer overlaps with the background noise by either increasing the amplitude of the vocalization to increase the signal‐to‐noise ratio and propagate further or by increasing the duration of the vocalization to provide a longer opportunity for perception (Wiley, 2013). However, we did not find a biologically significant change in vocalization parameters in this species of frog. Where significant change in frequency occurred, it was in the opposite direction as predicted, which would actually shift the calls further into the frequency range of road noise. The frequency change was not deemed to be biologically significant as the greatest amount of change was still within the standard deviation of centroid frequency. However, it is possible that this overall downward trend is evidence of a gradual, ongoing decrease in frequency that may result from unknown selective pressures. Lower‐pitched calls generally indicate fitter males in many species of frog, including *Acris crepitans* and *Hyla ebraccata* (reviewed in Chapter 10, Gerhardt & Huber, 2002). However, a downward frequency shift that increased the active space of a signal has been found in silvereyes (*Zosterops lateralis*). It is therefore possible that for this particular species of anuran, a decrease in frequency could decrease masking; more work is needed on the frequency and attenuation of this species. We did not find an overall lengthening of call duration or any increase in signal amplitude in response to noise, and the relationship between noise and call rate was not strong enough to be statistically significant. However, the downward trend of call rate in relation to increasing noise may be indicative of a meaningful shift. This may indicate a species in the beginning stages of changing their calls in some way, as high levels of traffic have only existed for the last 50 years (Kramer, 2004). However, it may also be that this species is unable to compensate in any way for increasing levels of background road noise.

**Figure 6:**
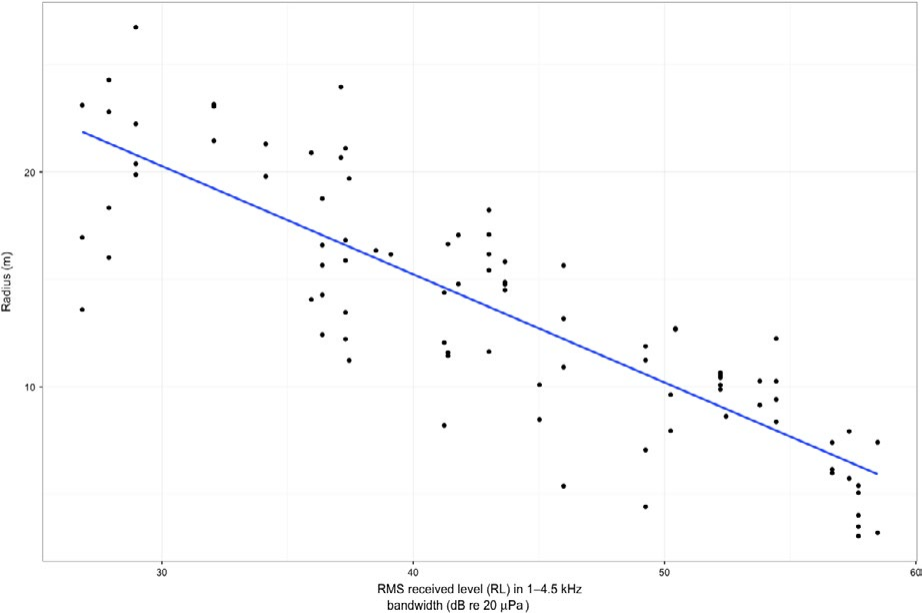
Linear mixed model (LMM) of the reduction in spatiotemporal communication radius with increasing levels of noise. The decrease in radius with increasing noise is significant at *p* < .005

**Figure 7:**
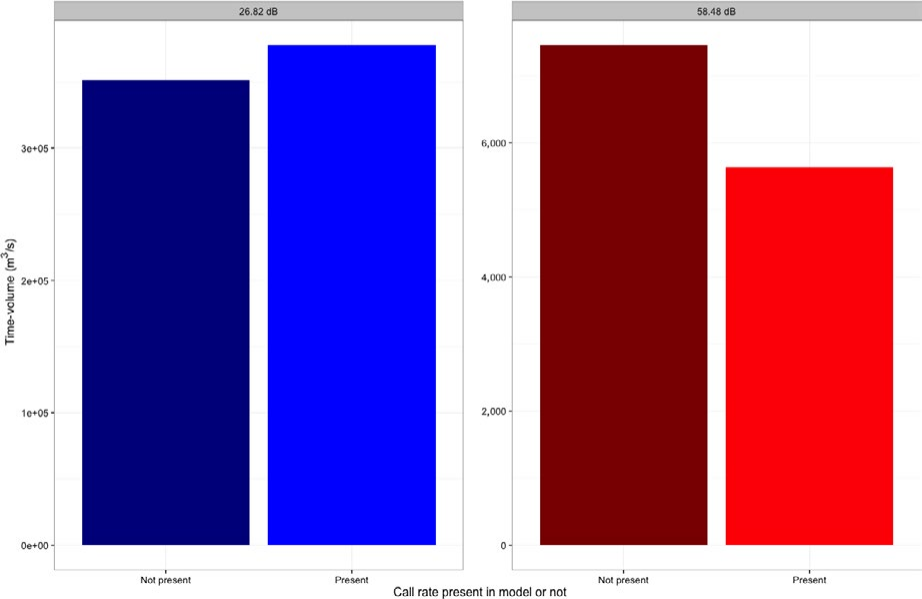
Difference in volume of spatiotemporal communication between the loudest recorded noise level (58.48 dB re 20 μPa) and the quietest (26.82 dB re 20 μPa, different scale on graph), as well as the change in time–volume if call rate is included in the model. Adding call rate to the model for the quietest noise level increased the communication time–volume by 7%, while adding it to the model for the loudest noise level decreased the communication time–volume by 32%

Communication is strongly impacted for male Pacific chorus frogs at noisier sites. For any given minute, our model indicates that spatiotemporal communication is drastically reduced for an individual Pacific chorus frog at sites with relatively high noise levels. In fact, when modeled against road noise and temperature, the radius of the hemisphere of spatiotemporal communication is reduced by 0.498 m for every 1 dB increase, which leads to a reduction in volume of 1.04 m^3^ per 1 dB increase (Figure 6). Thus, we have demonstrated that masking of advertisement calls by road noise in this species significantly impacts communication space. Additionally, if we include the downward trend of call rate in our model, we find that there is a substantial reduction in time–volume between the measure calculated without the call rate shift and that calculated with the call rate shift (Figure 7). This shift is much more dramatic for the loudest recorded road noise level, while at the quietest recorded noise level, the time–volume is actually increased because of the inclusion of call rate. The interaction between the temporal component of the time–volume and the noise level is not described by the traditional active space models. This emphasizes how important the temporal component of the spatiotemporal communication model can be for species that may rely on timing shifts. While this model represents an idealized situation, because it does not include vegetation, position of vocalizing frogs, temperature shifts, or substrate (Forrest, 1994), it can still inform our comparison of high‐noise versus low‐noise environments and how management intervention might optimize communication space and time for this species. It is likely that our model represents a best‐case scenario for this species of frog because it likely overestimates the radius of communication because of our low modeled detection threshold. Detection threshold, or the amplitude at which a signal is perceived, is usually well above the level of background noise in anurans (Vélez et al., 2013). For example, in work done on the confamilial species *Hyla chrysoscelis*, the threshold at which signals could be perceived above background chorus noise was 30 dB (Bee & Schwartz, 2009). If a similar threshold were found in Pacific chorus frogs, the radius found by our model would be even further reduced.

The reduction in spatiotemporal communication for this species and lack of any significant modification of calling parameters have significant ecological implications. Both communication and physiological impacts are possible for this species. As signals are perceived over a smaller area, serious consequences for mate attraction are possible. The signal reception by females is likely reduced because of the masking by road noise because of the increase in detection threshold level. This may alter females' ability to orient toward, and locate, calling males (Bee & Swanson, 2007). Additionally, anthropogenic noise may mask relevant cues within a call that are more likely to influence receiver behavior (Owren, Rendall, & Ryan, 2010), which may decrease signal recognition and/or alter female preference. For example, female *H. ebraccata* frogs preferred high‐frequency calls in the presence of moderate levels of noise and lost all preference when exposed to high levels of noise (Wollerman & Wiley, 2002). Both female and male anurans use the vocalizations of conspecifics to localize and orient toward breeding sites (Gerhardt & Huber, 2002). Compromised ability to localize and orient is compromised may have direct impacts on the capacity for frogs to breed successfully.

There may also be more direct physiological impacts from noise itself. Tennessen, Parks, and Langkilde (2014) found that female wood frogs (*Lithobates sylvaticus*) not only had reduced ability to orient in high noise situations, but also had increased levels of the stress hormone corticosterone. It is currently unknown how corticosterone affects breeding physiology and gamete health. However, raised levels of corticosterone have been found in amphibians attempting to cope with infection by *Batrachochytrium dendrobatidis* (Gabor, Fisher, & Bosch, 2013). Furthermore, it has been suggested that extremely elevated levels of corticosterone can inhibit reproduction in amphibians (Moore & Jessop, 2003).

In addition, we do not know how noise may interact synergistically with other stressors such as invasive species, disease, or overall habitat degradation to impact vocal amphibians. While it is possible that noise is not the most severe stressor to which amphibians are regularly exposed (McGregor et al., 2013), it may induce a similar stress response or exacerbate already degraded habitat. Recently, embryonic mortality and nest success were found to be deleteriously impacted by increased levels of road noise in captive zebra finches due to increased stress hormone production (Potvin & MacDougall‐Shackleton, 2015). Noise on its own can cause decreased body condition in migratory birds, without the compounding effects of habitat fragmentation caused by the road itself (Ware, McClure, Carlisle, & Barber, 2015). More work is needed to determine how noise interacts with other stressors and how this impacts vocalizing anurans.

### 4.1 Policy implications

Infrastructure improvements can aid in noise level reduction. The installation of sound barriers alongside particularly busy stretches of highway can buffer a considerable amount of noise, these can be constructed artificial building materials (Sanchez‐Perez, Rubio, Martinez‐Sala, Sanchez‐Grandia, & Gomez, 2002), or vegetation to create a semipermeable, natural barrier (Van Renterghem & Botteldooren, 2012). Advancements in environmentally friendly vehicle technology have also resulted in quieter cars (Komada & Yoshioka, 2014) that may have far‐reaching effects across other types of vehicles. Reductions in speed limits in critical habitat areas, such as near wildlife refuges, may result in decreased noise levels from transportation considerably.

Our model of spatiotemporal communication can quantify how additions of acoustic barriers would increase the communication space–time for this species. For example, pine tree vegetative barriers have been found to decrease the overall level of noise by 5 dB per 30.48 m of distance from the barrier (Van Renterghem, Botteldooren, & Verheyen, 2012). By placing this into the model for our noisiest site, the spatial radius of communication would be increased by 12–31.4 m. While this does not lower noise levels to those seen at sites far from highways, a doubling of communication radius is likely a considerable improvement for breeding Pacific chorus frog populations. Additionally, the amount of communication space returned to vocalizing frogs by the installation of acoustic barriers would vary by site and amplitude of the individual frogs.

This model of spatiotemporal communication is easily extensible to other terrestrial acoustic species. For a given species and site, the amount of time spent vocalizing as well as the space over which it can be perceived over background noise can be calculated, along with the insertion loss from a given type of acoustic barrier. However, the model requires perception data for the species in question as well as a noise threshold above which the communication space–time is too reduced for effective communication. Both perception and threshold will be species specific. Additionally, the model can be strengthened by the clarification of excess attenuation from other environmental factors such as vegetation and other acoustic scattering within the environment. With the inclusion of these factors, our model could become a valuable component of habitat suitability indices.

Animal communication is dependent upon the context that surrounds it, including the soundscape to which it contributes. Increasingly, this context is becoming dominated by anthropogenic noise. It is therefore integral to examine not only the spatial but also the temporal dimensions of animal communication to determine the extent to which it is impacted by the encroachment of noise. Our study determined that Pacific chorus frogs are not responding to traffic noise with changes to the spatial or temporal aspects of their call structure. Despite this, the space–time of an individual calling male frog is reduced in noisier habitats. Understanding how noise as a stressor impacts species such as the Pacific chorus frog, and modeling tangible ways that noise reduces their communication space–time and how that can be mitigated by noise barriers, gives us a valuable management tool to conserve our threatened natural soundscapes.

## Conflict of Interest

None declared.

## Note

1 This package is open‐source and in active development. It is available at github.com/dayvayen/commspacetime.
